# Enhancing Precision in Radiation Therapy for Locally Advanced Lung Cancer: A Case Study of Cone-Beam Computed Tomography (CBCT)-Based Online Adaptive Techniques and the Promise of HyperSight™ Iterative CBCT

**DOI:** 10.7759/cureus.66943

**Published:** 2024-08-15

**Authors:** Jingwei Duan, Joel A Pogue, Drexell H Boggs, Joseph Harms

**Affiliations:** 1 Radiation Oncology, University of Alabama at Birmingham, Birmingham, USA

**Keywords:** dosimetric analysis, cbct-dosimetry, tumor regression, synthetic ct, online-adaptive radiotherapy

## Abstract

This study explores the dosimetric benefits of cone-beam computed tomography (CBCT)-based online adaptive radiation therapy (oART) for a non-small-cell lung cancer (NSCLC) patient exhibiting significant tumor shrinkage during ChemoRT. The patient was prescribed 60 Gray (Gy) in 30 fractions and was initially treated with conventional RT. After the delivery of the first four treatment fractions, the patient’s treatment course was converted to oART due to tumor shrinkage seen on CBCT. Current oART dose calculations use a synthetic CT (sCT) image derived from deformable image registration (DIR) of the planning CT to the daily CBCT, and, as the tumor regressed, the discrepancy between the CBCT and the sCT increased, leading to a re-simulation after the delivery of the ninth fraction. In this case report, we first investigated dosimetric differences leveraged by converting this patient from conventional RT to oART. With oART using sCT, the patient's target coverage remained consistent with the reference plan while simultaneously changing lung V20 by 7.8 ± 1.4% and heart mean by 3.4 ± 1.5 Gy. Then, using this new simulation CT and comparing it with iterative CBCT (iCBCT) images acquired with the new HyperSight™ (HS) (Varian Medical Systems, Inc., Palo Alto, CA, USA) imaging system on the Ethos, we investigated the impact of direct dose calculation on HS-iCBCT as compared to sCT. The HS-iCBCT generated a dose distribution similar to the CT reference, achieving a 96.01% gamma passing rate using Task Group-218 (TG-218) criteria. Results indicate that HS-iCBCT has the potential to better reflect daily anatomical changes, resulting in improved dosimetric accuracy. This study highlights the advantages of oART in the presence of tumor response to therapy and underscores HS-iCBCT's potential to provide CT-level dose calculation accuracy in oART for NSCLC patients.

## Introduction

Radiation therapy (RT), combined with chemotherapy, is an effective treatment modality for inoperable non-small-cell lung cancer (NSCLC) patients [1]. Research has shown that high-dose radiation can lead to improved clinical outcomes in NSCLC patients, in both failure-free intervals and overall survival [2,3]. However, concerns have arisen regarding the increased exposure of nearby organs-at-risk (OARs) with escalated target doses, which may lead to a higher probability of normal tissue complications.

Adaptive radiation therapy (ART) has recently emerged as a promising solution for delivering high-conformity radiation doses to targets while minimizing doses to OARs by optimizing the treatment plan based on the patient's daily anatomy [4,5]. ART can reduce inter-fraction geometric uncertainties, thereby minimizing dosimetric discrepancies that arise from anatomical differences between simulation and treatment. 

Currently, synthetic computed tomography (sCT), generated from a deformable image registration (DIR) of the planning CT to the on-treatment cone-beam CT (CBCT), is the standard of care (SOC) for dose calculation in CBCT-based online ART (oART) using Ethos Treatment Planning System (TPS), version 1.1 [6,7]. Although oART with Ethos has been proposed as a feasible method for treating lung cancer [8,9], phantom studies have demonstrated that sCT has limitations in accurately reflecting density maps in the presence of substantial organ and patient changes [10,11]. Wegener et al. showed that errors in the sCT could lead to discrepancies between measured and delivered doses as high as 15% when the diameter of a circular target in a lung phantom changed from 3 to 1 cm [12]. The uncertainty in sCT-based dose calculations for lung cancer patients may be amplified by the significant heterogeneity between the tumor and surrounding tissue, which can substantially impact the overall quality of the treatment plan.

The recently introduced HyperSight™ (HS) (Varian Medical Systems, Inc., Palo Alto, CA, USA) imaging panel offers faster imaging acquisition times (six seconds) and CT-level imaging quality, enhanced by an iterative CBCT (iCBCT) reconstruction algorithm [1,13]. It is possible to perform direct dose calculation on HS-iCBCT instead of sCT, owing to its superior ability to reflect daily anatomy and improved Hounsfield unit (HU) accuracy. However, the impact of changing from calculation using sCT to HS-iCBCT in real lung patient cases remains unexplored but is a crucial prerequisite for clinical implementation. Herein, we describe an NSCLC case exhibiting significant tumor shrinkage during the treatment course of CBCT-based oART. This real patient case, characterized by significant tissue heterogeneity and tumor regression, demonstrated the dosimetric advantages of CBCT-based oART, using sCT, over traditional image-guided RT (IGRT). Furthermore, we compared the imaging performance of HS-iCBCT and sCT, highlighting their dosimetric differences in the context of significant tumor regression in real patient anatomy.

## Case presentation

Baseline patient and planning information

The patient is a 69-year-old gentleman with a history of coronary artery disease (CAD), a significant smoking history (48 years), and recently diagnosed right lung cT4N1 (invasion into the mediastinum) NSCLC, squamous cell carcinoma (SCC) subtype. Positron emission tomography (PET)/CT revealed significant avidity within the right middle lobe mass (5.8 cm, maximum standardized uptake value 9.3), consistent with malignancy. This was associated with post-obstructive atelectasis. There was no evidence of distant metastatic disease or hypermetabolic adenopathy outside of an adjacent right hilar node. A biopsy of the right hilar mass/11R revealed NSCLC, morphologically compatible with SCC (4L, 7, 4R benign). The patient was evaluated by thoracic surgery and was found not to be a surgical candidate given the extent of disease and poor pulmonary function. Repeat CT of the chest, six weeks after the evaluation PET, demonstrated interval enlargement of the tumor to 6.2 x 5.9 cm and development of post-obstructive atelectasis around the mass due to interval obstruction of the right middle lobe bronchus and right upper lobe subsegmental bronchi. For initial planning, the gross tumor volume (GTV) was identified using CT simulation. The clinical target volume (CTV) was expanded by 7 mm from the GTV, with 5 mm to account for subclinical disease and 2 mm to account for internal margins, as the patient was scanned under mid-respiratory cycle breath-hold but treated free-breathing. A further 5 mm expansion from the CTV was used to derive the planning target volume (PTV). The treatment plan was optimized and calculated by the dedicated dosimetrist on Ethos TPS v1.1.

Timeline of treatment

**Figure 1:**
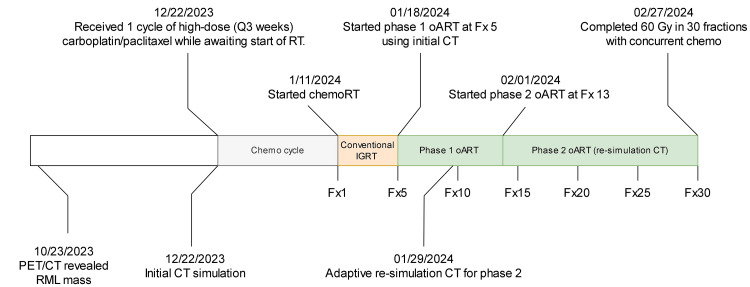
Patient’s diagnosis and treatment timeline. oART: Online adaptive radiation therapy; PET/CT: Positron emission tomography/computed tomography; RML: Right middle lobe; Fx: Fraction; IGRT: Image-guided radiation therapy

The timeline of this patient's treatment is illustrated in Figure 1. The patient started one cycle of high-dose (every three-week dose) carboplatin/paclitaxel and began RT during the second cycle of chemotherapy. The radiation treatment was prescribed at 60 Gy in 30 fractions, with concurrent weekly chemotherapy consisting of carboplatin/paclitaxel. The patient was initially simulated using supine four-dimensional CT (4DCT), with arms overhead, and immobilization devices including an Alpha Cradle and Knee Fix. Tumor motion, as assessed by the 4DCT, was measured as less than 5 mm in all directions. For the initial plan target contouring, a maximum intensity projection (MIP) image was generated using phases 0% to 90% of the respiratory cycle. The averaged 4DCT, also incorporating phases 0% to 90%, was utilized for organ contouring and treatment planning. The initial treatment plan was delivered with conventional IGRT on the Ethos, from fractions 1 to 4.

**Figure 2:**
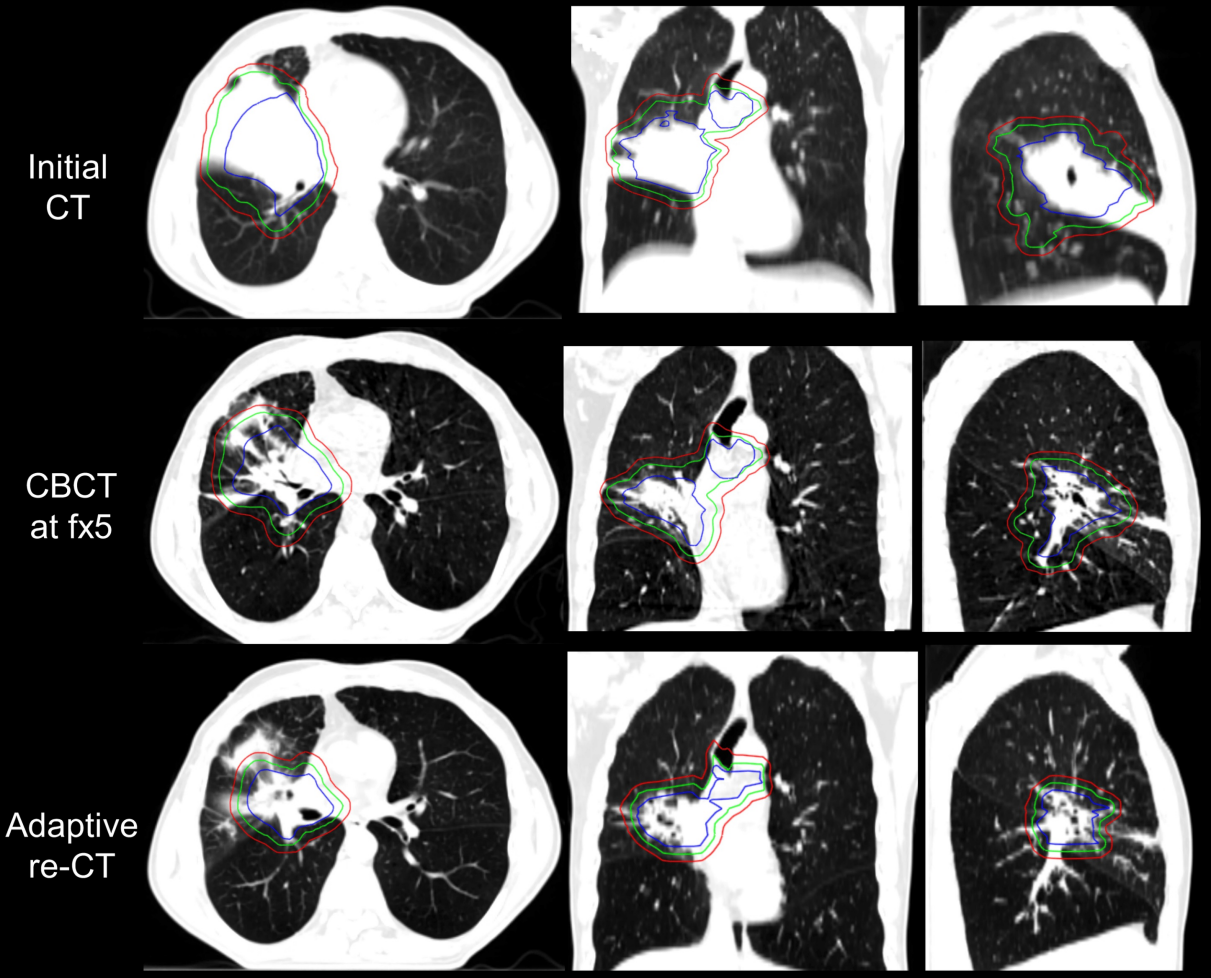
Three views of the patient’s CT simulations at different times. The blue, green, and red contours indicate the GTV, CTV, and PTV, respectively. GTV: Gross tumor volume; CTV: Clinical target volume; PTV: Planning target volume; CBCT: Cone-beam computed tomography; Fx: Fraction

As noted on daily imaging, the PTV volume decreased by 54.48% between the initial planning CT (December 22, 2023, 782.1 cc) and the start of treatment (January 11, 2024, 356.0 cc). This volume reduction may be attributed to the high-dose chemotherapy cycle administered prior to radiation treatment, as Figure 1 indicates. The significant target regression motivated the shift from conventional IGRT to oART. Based on this tumor volume change, the treatment plan was revised to CBCT-based oART, starting from fraction 5. Of note, the sCTs for the first phase of oART were generated through DIR of the initial sim CT to the on-treatment CBCT. Due to the observed limitations of sCT, the clinical team decided to perform a re-CT for this patient. Subsequently, a new treatment planning inspiration breath hold (INBH) CT (adaptive re-simulation) was taken on January 29, 2024, after delivery of the patient’s ninth treatment fraction, due to continued tumor volume and shape changes. This second INBH CT will be referred to as the re-CT from here on. The target and organs were contoured, and the plan was optimized on the re-CT. After going through appropriate quality assurance steps, this re-CT scan was used as the reference CT scan from fraction 13 through the end of treatment. As part of this adaptive planning revision, the original treatment plan and optimization template were not modified, only the reference CT image. This report will focus on oART from fraction 5 to fraction 30, the end of treatment. Figure 2 illustrates the initial planning CT, the CBCT at fraction 5 (day 1 in phase 1 oART treatment), and the re-CT.

oART plan

In this study, the thorax six-second HS-iCBCT was utilized, with an X-ray tube voltage of 125 kVp and auto-exposure (203 mAS) for CT dose modulation. The field of view (FOV) of HS-iCBCT was determined during the first oART fraction by therapists using a digitally reconstructed radiograph (DRR) image with target contours. The FOV was set to 24 cm to ensure it encompassed the target and surrounding tissues. Importantly, the patient was instructed to hold his breath in the middle of the respiratory cycle during the six-second CBCT acquisition, to minimize any motion artifacts in the HS-iCBCT. During oART treatment, the same margin receipt was used as described in the previous section. The patient was scanned under mid-respiratory cycle breath-hold but treated free-breathing. Intra-fraction motion management was conducted using a surface-guided monitoring system [14]. Following the standard workflow for oART, the target and OAR contours were generated from the on-treatment HS-iCBCT and transferred to the sCT before modification and final approval by the treating physicist. For each daily oART fraction, a nine-field intensity-modulated RT (IMRT) adaptive plan was optimized and calculated using daily sCT. Those operations were conducted by the dedicated oART team in our institution, the workflow of which has been detailed in a previous study [15]. An open-source plan scorecard was used to assess individual plan quality across various images and their associated daily contours [16]. This scorecard employs multiple piecewise linear scoring functions, derived from our institutional lung treatment team consensus, to evaluate both target coverage and OAR sparing. The plan score ranges from 0 to 100, with higher scores indicating better plan dosimetric quality.

Adaptive dosimetric benefits

After oART treatment, the session data from oART (fx5-fx30), including HS-iCBCT and sCT images, daily contours, and treatment plans (adaptive and scheduled, i.e., initial plan recalculated on sCT), were transferred from the Ethos TPS to the Eclipse TPS (Varian Medical Systems, Inc., Palo Alto, CA, USA) for three reasons: (I) calculation of HS-iCBCT plans, (II) re-calculation of sCT plans, and (III) utilization of the built-in Eclipse scripting interface for dose-volume histograms (DVHs) analysis. All plans used in this study were calculated using Acuros XB (v 16.10) in both Ethos and Eclipse. sCT plans were recalculated in Eclipse because of subtle differences between Eclipse and Ethos voxel discretization. While these differences are expected to be minimal, calculating plans using HS-iCBCT and sCT with a single TPS enabled a direct comparison. For each fraction, the dose was scaled to the full course dose.

**Figure 3:**
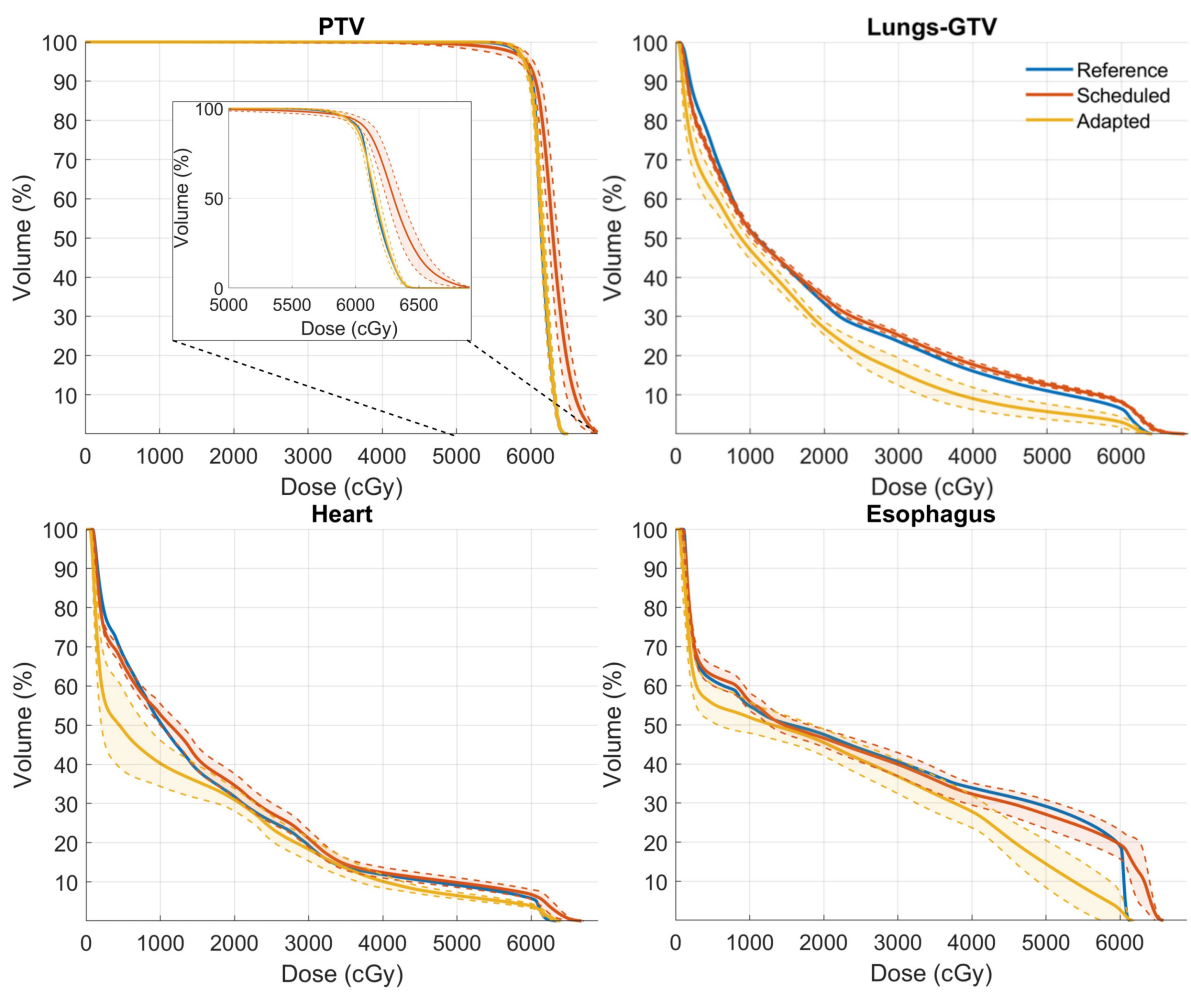
Mean DVHs for the reference, adapted, and scheduled plans. The scheduled and adapted plans were both calculated on sCT for each oART session. The shaded areas represent the standard deviation across 26 fractions. Each individual fraction dose was scaled to the full-course dose before averaging. DVHs: Dose-volume histograms; sCT: Synthetic computed tomography; oART: Online adaptive radiation therapy; PTV: Planning target volume; GTV: Gross tumor volume

**Figure 4:**
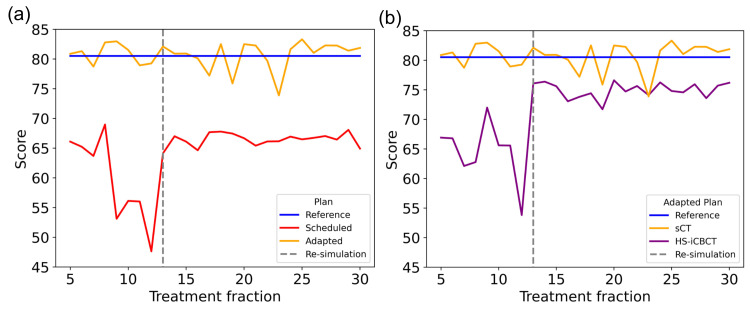
a) The plan scores of the scheduled plan and adapted plan, both calculated on sCT; b) The plan scores of the adapted plan calculated on HS-iCBCT and sCT, respectively. sCT: Synthetic computed tomography; HS-iCBCT: HyperSight iterative cone-beam computed tomography; oART: Online adaptive radiation therapy

The dosimetric benefits of CBCT-based oART were analyzed by comparing two scenarios for the oART treatment session: the daily adaptive plan optimized and calculated on sCT, and the scheduled plan re-calculated on sCT, both baselined against the reference plan calculated on the initial CT. In this analysis, sCT was used for dose calculation and optimization, as it represents the current SOC. DVHs were exported from each plan and are shown in Figure 3, with solid lines and shaded areas illustrating the mean and standard deviation among the treatment courses, respectively. The mean PTV D98% improved by 3.6 ± 3.8%, while the mean PTV V105% was reduced by 36.7 ± 10.1%. For OARs, the volume of the lungs-GTV receiving 20 Gy (V20Gy) was reduced by 7.8 ± 1.4%, the esophagus mean dose (Dmean) was reduced by 4.4 ± 2.2 Gy, and the heart Dmean was reduced by 3.4 ± 1.5 Gy. The plan scores of these two scenarios are demonstrated in Figures 4a-4b. Adaptive plans calculated on sCT consistently matched the quality of reference plans on initial CT, while scheduled plans consistently scored around 15-20 points lower. These results demonstrate the potential benefits of adaptive planning in improving target coverage while simultaneously reducing doses to critical OARs.

Direct dose calculation comparison

With the upcoming release of Ethos 2.0, the default option for online optimization and dose calculation will transition from the sCT to the HS-iCBCT. Research has shown that the median HU value in breath-hold 125 kVp HS-iCBCT is within 15 HU of that in CT simulation for muscle, fat, bone, and lung tissue types, indicating the utility of HS-iCBCT for direct dose calculation by using the CT calibration curve within an acceptable range [17].

**Figure 5:**
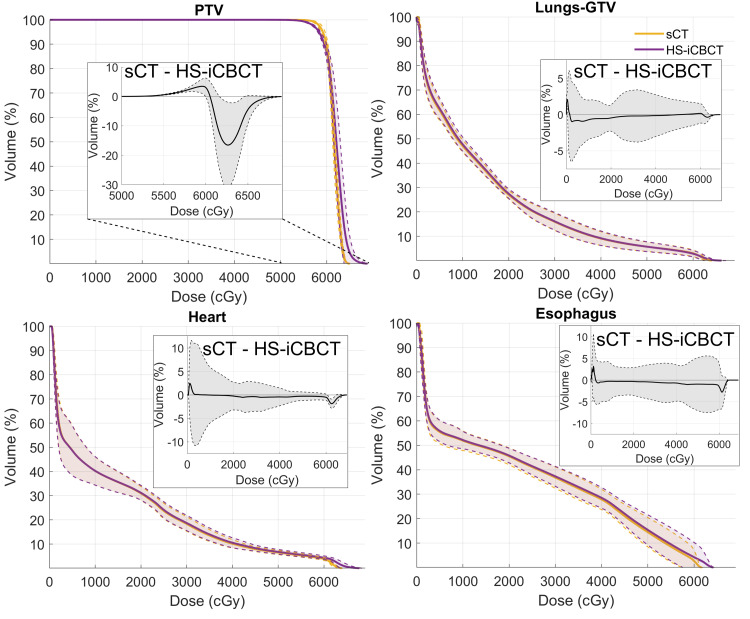
Mean DVHs of adapted plans calculated on HS-iCBCT and sCT for all oART sessions. The differences between sCT and HS-iCBCT calculations (sCT minus HS-iCBCT) are inserted. The shaded area indicates the standard deviation among 26 fractions. Each individual fraction dose is scaled to the full-course dose before averaging. DVHs: Dose-volume histograms; HS-iCBCT: HyperSight iterative cone-beam computed tomography; sCT: Synthetic computed tomography; oART: Online adaptive radiation therapy; PTV: Planning target volume; GTV: Gross tumor volume

To evaluate the impact that this change will have on calculated dose distributions in the lung, we carried out a retrospective analysis where the adapted plans were recalculated directly on the HS-iCBCT images and compared to the sCT images, which had been generated during oART. Figure 5 shows the mean DVH (calculated across all 26 adaptive fractions) from adaptive plans optimized and calculated using the sCT, and the same plans re-calculated using the HS-iCBCT directly. The insets illustrate the residuals between plans calculated using sCT and HS-iCBCT, showing that, although low doses were similar, the PTV heterogeneity was significantly higher when using HS-iCBCT for calculation instead of sCT. Note that the adapted plans were optimized on sCT during oART. Adapted plan quality scores on HS-iCBCT and sCT are shown in Figure 4b. The adapted plan scores, calculated on HS-iCBCT, showed improvement following re-CT at the 13th fraction. This comparison highlights that, while there are noticeable discrepancies between HS-iCBCT and sCT calculations, the overall benefits of adaptive planning remain significant.

To thoroughly analyze the dosimetric differences fraction-by-fraction, a comparison between the dose calculations from HS-iCBCT and sCT was carried out using gamma evaluation. The criteria for gamma evaluation included 1%/1 mm and 3%/2 mm tolerances for global dose difference/distance to agreement (DTA), with dose thresholds set at 10% and 80%. The criteria of 3%/2 mm and a 10% dose threshold were based on Task Group-218 (TG-218) guidelines, while the other criteria were employed to highlight discrepancies with the strictest criteria (1%/1 mm) or in high-dose areas (80% threshold) [18].

**Figure 6:**
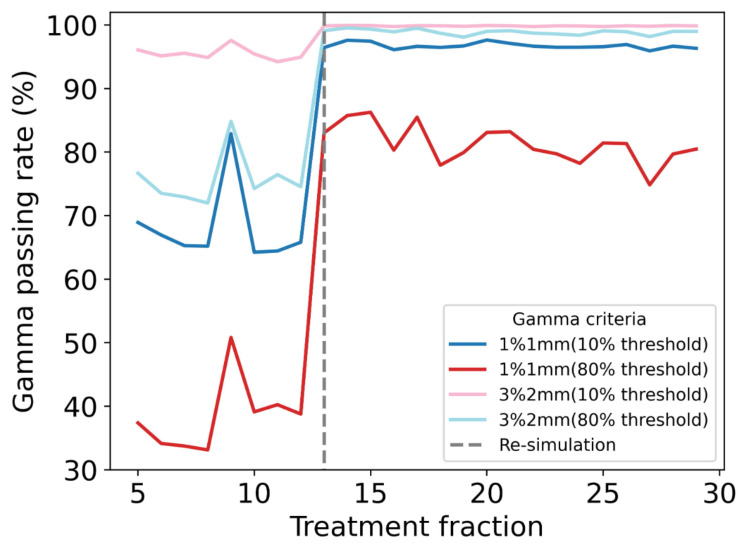
Gamma passing rate between HS-iCBCT and sCT throughout the oART treatment course under different criteria. HS-iCBCT: HyperSight iterative cone-beam computed tomography; sCT: Synthetic computed tomography; oART: Online adaptive radiation therapy

The gamma passing rate over time, given different criteria, is shown in Figure 6. During phase 1 of oART (fractions 5-12), the gamma passing rate was 95.47 ± 1.01% for 3%/2 mm criteria and 67.96 ± 6.22% for 1%/1 mm criteria with a 10% threshold, showing modest agreement between the sCT and the HS-iCBCT. However, during phase 2 of oART (fractions 13-30), where the re-CT was used as the baseline for sCT generation, the gamma passing rate improved to 99.85 ± 0.06% for 3%/2 mm and 96.72 ± 0.48% for 1%/1 mm with a 10% threshold. Similar improvements were observed in the high-dose region (80% threshold), where the gamma passing rate improved from 75.65 ± 4.05% to 98.89 ± 0.41% for 3%/2 mm, and from 38.41 ± 5.69% to 81.24 ± 2.99% for 1%/1 mm. Additionally, the minimum passing rate improved in phase 2 oART, where the baseline of sCT generation was changed. For the 3%/2 mm criterion with a 10% threshold (TG-218 scope), the minimum improved from 94.22% in phase 1 to 99.74% in phase 2. For the 3%/2 mm criterion with an 80% threshold, the minimum passing rate rose from 71.98% in phase 1 to 98.08% in phase 2.

**Figure 7:**
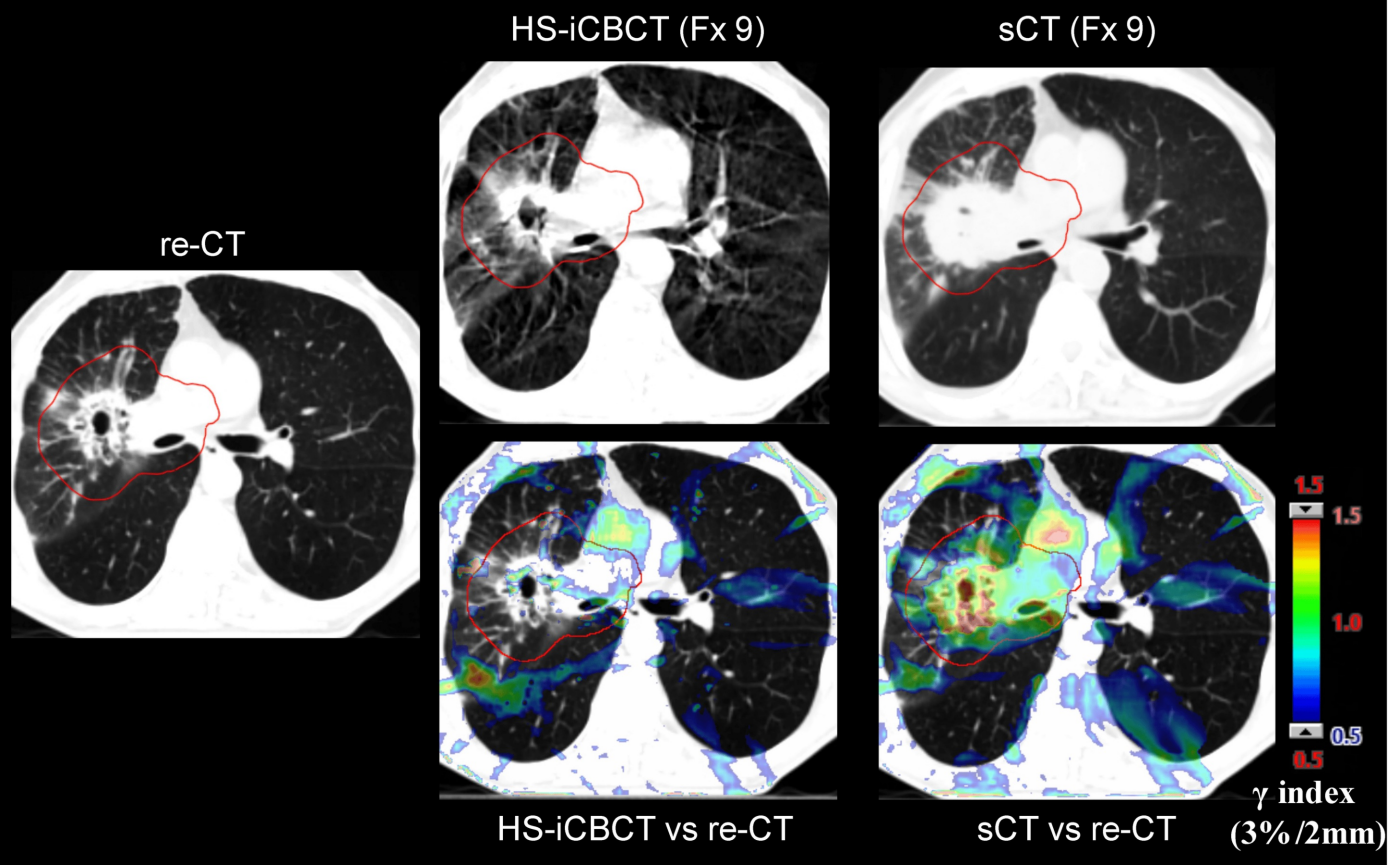
Images from the re-CT, along with HS-iCBCT and sCT at fraction 9, are presented. Using re-CT as the reference, the gamma distributions of HS-iCBCT and sCT with a criterion of 3%/2 mm are shown on re-CT. HS-iCBCT: HyperSight iterative cone-beam computed tomography; sCT: Synthetic computed tomography; Fx: Fraction

As shown in Figure 1, the re-CT was acquired on the day of fraction 9 after treatment, at which point the initial CT was still being utilized for sCT generation. Given that the re-CT and ninth fraction HS-iCBCT were taken hours apart, the re-CT was treated as the ground truth for comparing the dose distributions from HS-iCBCT and sCT at the ninth fraction, using the daily adaptive plan created for that fraction. The sCT images were generated through DIR of the planning CT to HS-iCBCT. As a result, the sCT and HS-iCBCT shared the same frame of reference. The HS-iCBCT and sCT images were rigidly registered to the re-CT to ensure they shared the same frame of reference, and gamma analysis was performed, with the results shown in Figure 7. Using criteria of 3%/2 mm and a 10% dose threshold, the calculation using the HS-iCBCT achieved a 96.01% passing rate with a mean gamma of 0.37, while sCT achieved a 91.94% passing rate with a mean gamma of 0.45. At an 80% threshold, HS-iCBCT maintained high performance with a 91.83% passing rate and a mean gamma of 0.44, whereas sCT performance decreased to a 72.75% passing rate with a mean gamma of 0.72.

These results demonstrate that a plan calculated on HS-iCBCT produced a dose distribution comparable to that calculated on the re-CT reference in the presence of significant tumor shrinkage, while the calculation on the sCT did not agree with the planning CT. Based on these findings, it is reasonable to assume that the HS-iCBCT can more closely reflect the real patient anatomy in other fractions compared to sCT. Consequently, dose distributions calculated on HS-iCBCT for adapted plans may more closely reflect the true dose delivery to the patient. The plan scores and gamma passing rates, as seen in Figure 4b and Figure 6, showed stronger agreement between sCT and HS-iCBCT in phase 2 of oART than in phase 1, indicating that the sCT had more difficulty representing large changes in underlying anatomy. Based on the limitations of sCT observed in anthropomorphic phantom studies [10] and the findings from this case study involving significant tumor shrinkage, the authors believe that the sCT is unable to provide an accurate density map for this patient due to significant anatomical changes during phase 1 of oART (fractions 5-12), which significantly impacted dose calculation and thereby affected the adaptive plan optimization based on sCT. This highlights the potential benefits of using updated imaging data (re-CT) for more accurate dose calculation and adaptive planning when relying on sCT, especially in cases with significant anatomical changes during the treatment course.

## Discussion

This case report describes the treatment of a patient with NSCLC who experienced atelectasis prior to simulation, followed by significant tumor shrinkage during the course of chemoradiation. CBCT-based oART offered a flexible solution that allowed the treatment team to continually monitor and adapt to anatomical changes during treatment, providing substantial dosimetric benefits compared to non-adaptation, as illustrated in Figure 3. However, the current implementation of oART comes with some degree of uncertainty in dose delivery due to the use of sCT for plan optimization and calculation, particularly in the presence of large anatomical changes. These uncertainties are problematic because, in theory, patients with these changes during treatment have the highest potential clinical benefit from oART. Nonetheless, the authors believe that the dosimetric benefits of oART still outweigh the uncertainties introduced by sCT.

A significant anatomy change can be observed between the initial and re-CT, as shown in Figure 2. This case study highlighted the limitation of sCT in accurately reflecting density maps in a patient with significant anatomical changes, resulting in significant dosimetric inaccuracies. Considering that plan optimization is also currently performed using sCT on the Ethos TPS (v1.1), this raises the possibility of sub-optimal oART plan quality due to the potential for optimization being driven by inaccurate density maps, as illustrated in Figure 7. Therefore, the authors suggest exercising additional caution when adapting treatment plans in cases where sCT may be unreliable. A practical approach to mitigate these uncertainties is to perform a re-CT in the presence of tumor change, which serves as a more accurate reference for sCT generation.

Another promising alternative solution to address uncertainties from sCT is to perform direct dose calculation on HS-iCBCT. The HS platform's hardware development offers several advantages over its predecessor, including an enlarged FOV, reduced motion artifacts, and improved HU accuracy [1,13,17]. HS-iCBCT shows promising potential as an imaging modality that not only captures daily anatomical changes but also provides comparable performance for direct dose calculation. These features make it an attractive option for CBCT-based oART and may help address the limitations of sCT mentioned previously. The authors recognize that results from a single patient do not provide conclusive evidence that HS-iCBCT is generally more accurate than sCT, and thus, the results here should not be applied to other patients and sites. Rather, the purpose of this study is to highlight differences between sCT and HS-iCBCT that Ethos users should be aware of, which can have large effects on dose accuracy in the presence of significant heterogeneity. It remains the responsibility of individual clinics to use their discretion in selecting sCT or HS-iCBCT for use until dosimetric investigations across a broader range of sites and patient populations are conducted to further validate the dosimetric findings in this case study.

Large anatomical changes during treatment present a challenge for interfraction contouring consistency. In the oART workflow, the GTV contours are propagated from the planning CT to the on-treatment CBCT according to the deformation vector fields from the DIR. When there are large deformations or inaccuracies in the registration, the GTV contours may need substantial edits, which introduces room for error, especially when the primary adaptor changes between fractions. For this patient, the average GTV contour volume increased from 124 ± 16 cm³ during fractions 5-8 to 191 ± 15 cm³ for fractions 9-12, likely contributing to the decrease in plan scores before re-simulation, as shown in Figures 4a-4b. While the physicist is responsible for contouring on-treatment in our institution, an attending radiation oncologist also performs an offline review of each adaptive fraction. The large discrepancy between these two fraction blocks indicates that both the physicist and radiation oncologist had difficulty delineating the target volume prior to re-simulation. These results suggest that even though direct dose calculation with HS-iCBCT may address the technical limitations of sCT, the re-CT process, including delineation of target contours on the new image set, may still be necessary to ensure high-quality adaptive treatment and to reduce inter-fraction and inter-observer variability during oART. This also demonstrates the need for real-time quality assurance, both for contour and for plan quality, during adaptive treatment sessions.

As radiation technology evolves, proton therapy, renowned for its precision, can improve clinical outcomes for various tumors [19,20], with potential additional benefits stemming from adaptive therapy [21]. Nevertheless, interfraction motion remains a concern in proton therapy (particularly in the lung), and there remain substantial differences between photon and proton treatments. Thus, more research is needed to develop and evaluate adaptive strategies specific to proton therapy and to quantify dosimetric benefits.

## Conclusions

This study found notable dosimetric benefits using CBCT-based oART for the treatment of a patient with NSCLC who experienced tumor regression during the course of treatment. Notably, the patient was treated shortly after HS-iCBCT was incorporated into our clinical workflow. The sCT currently used for oART was unable to accurately model the large anatomical changes that the patient experienced, leading to potential discrepancies between dose calculation and delivered dose. The HS-iCBCT better reflected these anatomical changes, and a re-simulation CT taken in the middle of this patient’s treatment course allowed for the evaluation of both the sCT and HS-iCBCT against a planning CT. Dose calculation on the HS-iCBCT more closely agreed with the planning CT, indicating that the use of HS-iCBCT may be more appropriate for treatment plan optimization and calculation than sCT.

## Appendices

**Table 1:** Abbreviation and description of terms used in this study.

Abbreviation	Full term	Description	Usage
CT	Computed tomography	Standard diagnostic imaging technique using X-rays to create cross-sectional images	Used as a reference
HS-iCBCT	HyperSight iterative cone-beam computed tomography	HyperSight CBCT with iterative reconstruction algorithm	Used for imaged guided radiation treatment, daily contour generation, sCT generation, and adaptive plan dose calculation
sCT	Synthetic computed tomography	sCT images generated from the Ethos platform (V1.1) through a series of steps starting with deformable image registration (DIR) of the planning CT (pCT) to the on-treatment CBCT	Used for adaptive plan optimization and dose calculation
